# Therapeutic potential of folic acid supplementation for cardiovascular disease prevention through homocysteine lowering and blockade in rheumatoid arthritis patients

**DOI:** 10.1186/s40364-015-0049-9

**Published:** 2015-09-04

**Authors:** Mickael Essouma, Jean Jacques N. Noubiap

**Affiliations:** Division of Medicine, Sangmelima Referral Hospital, P.O. Box 890, Sangmelima, Cameroon; Department of Medicine, Groote Schuur Hospital and University of Cape Town, Cape Town, South Africa; Medical Diagnostic Center, Yaoundé, Cameroon

**Keywords:** Rheumatoid arthritis, Cardiovascular diseases, Prevention, Inflammatory biomarkers, Hyperhomocysteinemia, Folic acid supplementation

## Abstract

Rheumatoid arthritis (RA) is a chronic inflammatory disease that preferentially affects joints, and characterized by an approximately two-fold increased risk of cardiovascular diseases compared with the general population. Beyond classical cardiovascular risk factors, systemic inflammatory markers are primarily involved. Hence, anti-inflammatory strategies such as homocysteine-lowering interventions are warranted. Indeed, hyperhomocysteinemia is commonly found in RA patients as a result of both genetic and non-genetic factors including older age, male gender, disease-specific features and disease-modifying antirheumatic drugs. Most importantly in the pathophysiology of hyperhomocysteinemia and its related cardiovascular diseases in RA, there is a bi-directional link between immuno-inflammatory activation and hyperhomocysteinemia. As such, chronic immune activation causes B vitamins (including folic acid) depletion and subsequent hyperhomocysteinemia. In turn, hyperhomocysteinemia may perpetrate immuno-inflammatory stimulation via nuclear factor ƙappa B enhancement. This chronic immune activation is a key determinant of hyperhomocysteinemia-related cardiovascular diseases in RA patients. Folate, a homocysteine-lowering therapy could prove valuable for cardiovascular disease prevention in RA patients in the near future with respect to homocysteine reduction along with blockade of subsequent oxidative stress, lipid peroxidation, and endothelial dysfunction. Thus, large scale and long term homocysteine-lowering clinical trials would be helpful to clarify the association between hyperhomocysteinemia and cardiovascular diseases in RA patients and to definitely state conditions surrounding folic acid supplementation. This article reviews direct and indirect evidence for cardiovascular disease prevention with folic acid supplementation in RA patients.

## Background

Rheumatoid arthritis (RA) is a systemic inflammatory disease characterized by chronic symmetric and erosive synovitis that preferentially affects peripheral joints. RA patients have an approximately two-fold increased risk of myocardial infarction, cerebrovascular events and deep venous thrombosis as well as a 60 % increased risk for cardiovascular diseases (CVD) compared with the general population [1–3]. Irrespective of traditional cardiovascular risk factors, systemic inflammatory mediators characteristic of RA are primarily involved [1].

Homocysteine is a sulfhydryl-containing amino acid mainly formed from the essential amino acid methionine. Its plasma concentration depends on age, sex, lifestyle factors-(coffee consumption, smoking, physical activity, alcohol)-, genetic mutations leading to a severely diminished activity of the enzymes involved in homocysteine catabolism, drugs and diseases interfering with its metabolism, and most importantly with B vitamins intake. Since B-group vitamins (folic acid, pyridoxine and cobalamine) are involved in homocysteine catabolism, their plasma levels are inversely associated with that of homocysteine [4]. Thus, hyperhomocysteinemia (HHcy) which refers to fasting plasma homocysteine concentrations ≥ 15 μM [4], is associated with decreased folate levels. HHcy is a well-known cardiovascular risk factor in the general population [4, 5], and in RA patients [6, 7].

Considering the CVD epidemic in RA patients and the important role of systemic inflammation, anti-inflammatory strategies such as homocysteine-lowering interventions appear necessary. This paper reviews direct and indirect evidence for CVD prevention with folic acid supplementation in RA patients.

## Hyperhomocysteinemia in rheumatoid arthritis

**Fig. 1:**
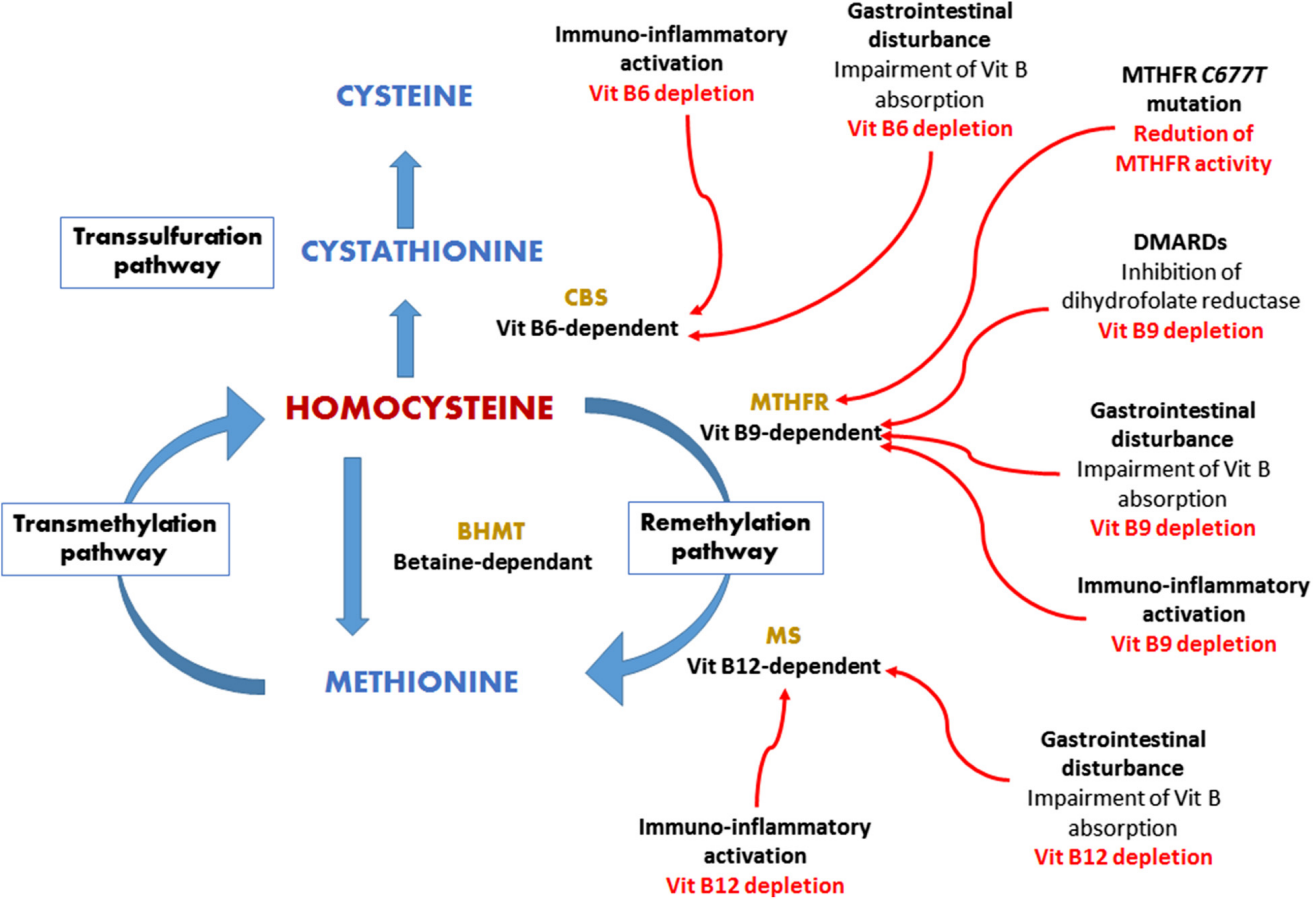
Homocysteine metabolism and major factors associated with hyperhomocysteinemia in rheumatoid arthritis patients. MTHFR methylene tetrahydrofolate; CBS cystathione β synthase; BHMT betaine homocysteine methyltransferase; MS methionine synthase; Vit vitamin; DMARDs disease-modifying antirheumatic drugs. Vit B9 depletion owing to immuno-inflammatory activation, DMARDs, and gastrointestinal disturbance impairs the MTHFR vit B9-dependent remethylation pathway together with the MTHFR C677T mutation; vit B12 depletion owing to both immuno-inflammatory activation and gastrointestinal disturbance impairs the MS vit B12-dependent remethylation pathway; vit B6 depletion owing to immuno-inflammatory activation and gastrointestinal disturbance impairs the CBS vit B6-dependent transsulfuration pathway

HHcy is common among RA patients, as a consequence of both genetic and non-genetic factors associated with the disturbance of homocysteine metabolism [7–22]. Genetic risk factors are essentially represented by the methylenetetrahydrofolate reductase (MTHFR) 677C > T homozygous or heterozygous genotype which results in impaired homocysteine methylation to form methionine [8]. Whereas non-genetic factors include older age [9, 10], male gender [9, 10], RA-specific features [4, 7, 11–22], and disease-modifying antirheumatic drugs (DMARDs) [23–28] (Fig. 1).

RA-specific features that influence the development of HHcy are mainly immuno-inflammatory activation together with extra-articular features (e.g., gastrointestinal disorders and kidney dysfunction), and their resultant B vitamins (folic acid, pyridoxine and cobalamine) deficiency [4, 7, 11–20]. Antiphospholipid (aPL) autoantibodies could contribute to a lesser extent [21, 22]. In particular, CD4^+^CD28^−^ cells found in RA patients produce interferon gamma (IFN-γ) [11–13]. The latter cytokine activates T helper 1 (Th1) cells that spill over pro-inflammatory cytokines (tumor necrosis factor alpha [TNF-α], interleukin-1 [IL-1], interleukin-6 [IL-6]) [11, 13]. These cytokines perpetually stimulate excess monocyte/macrophage production of reactive oxygen species (ROS) free radicals, thus causing cellular damage [11]. The net result is a long-term shift in immuno-inflammatory activation, with antioxidant enzymes overloaded by excess ROS [11–13]. Then, other oxidation-sensitive molecules such as B vitamins (including folate) are targeted by ROS [11], and rapid synthesis of deoxyribonucleic acid (DNA) occurs within immunocompetent cells [14]. Hence, both oxidative stress and increased mitosis account for B vitamins deficiency. This deficiency could be exacerbated by gastrointestinal disturbances [15–20]. Indeed, B vitamins are absorbed across the small and large intestinal mucosa through active transport and passive diffusion [15–19]. Concerning folic acid absorption, the dietary vitamin B9 is transported by the mucosal proton-coupled folate-transporter across the apical brush-border membrane of the bowel, whereas the vitamin B9 from commensal bacteria is predominantly absorbed in the colon [15, 16]. However, RA is associated with frequent occult gut inflammation (due to the combination of both intestinal immune activation and the use of anti-inflammatory agents) which alters the mucosal structure and permeability [15, 19]. Furthermore, decremented intestinal microbiota has been reported in RA patients [20]. Hence, gastrointestinal disorders may reduce folic acid uptake in RA populations. In turn, folic acid deficiency along with vitamins B_6_ and B_12_ deficiencies cause homocysteine accumulation, given that they are co-factors for homocysteine catabolism [4, 7]. Homocysteine may further accumulate in RA patients with co-morbid kidney dysfunction since kidneys are key organs for the metabolism of homocysteine [4]. On the other hand, Seriolo and collaborators observed significantly incremented serum homocysteine concentration in aPL-positive female RA patients compared to their aPL-negative RA counterparts and to non-RA controls (16.6 ± 5.6 mol/l vs 13.9 ± 5.1 mol/l and 9.3 ± 4.1 mol/l, respectively; *p* <0.01 and *p* <0.0001) [21]. This finding together with the high prevalence of aPL autoantibodies (28 %) in RA patients [22] is suggestive of the role of aPL in the elevation of plasma homocysteine in RA patients, but the mechanistic link is still obscure.

DMARDs, essentially methotrexate, cause HHcy via folate depletion [23–28]. This requires inhibition of dihydrofolate reductase-an enzyme involved in homocysteine remethylation pathway [8, 23, 24]. Noteworthy, the effect of methotrexate is exacerbated when it is combined with sulfasalazine [13], or in patients exhibiting the MTHFR 677C > T genotype [8].

## Association between hyperhomocysteinemia and cardiovascular diseases

### In the general population

**Fig. 2:**
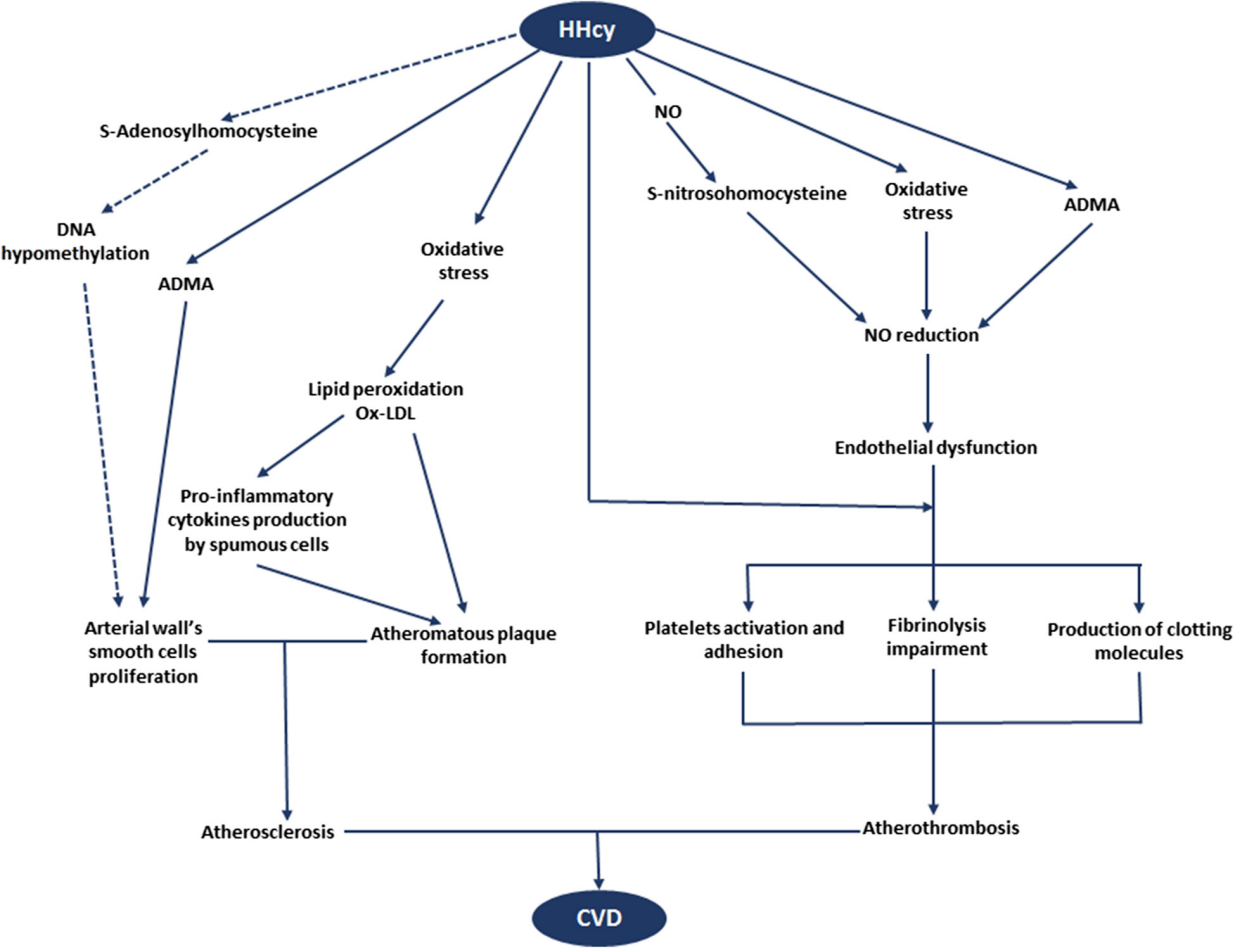
Mechanisms explaining homocysteine-related cardiovascular diseases at large. Dark arrow main mechanisms; dotted arrow minor mechanism. HHcy Hyperhomocysteinemia; NO nitric oxide; DNA deoxyribonucleic acid; ADMA asymmetric dymethyl arginine; ox-LDL oxidized low density lipoprotein cholesterol; CVD cardiovascular diseases. Through S-nitrosohomocysteine, ADMA and oxidative stress, HHcy reduces NO bioavailability, thus causing endothelial dysfunction. Under high propensity for coagulation (characterized by platelet adhesion and activation, production of clotting molecules, impaired fibrinolysis) that can be exacerbated by HHcy, endothelial dysfunction evolves towards atherothrombosis. Besides, HHcy-related oxidative stress increases ox-LDL production hence leading to formation of the atheromatous plaque which together with arterial smooth muscle cells proliferation trigger atherosclerosis. Atherosclerosis and atherothrombosis (completed atherosclerosis with ruptured plaque and thrombosis) lead to CVD

HHcy is independently associated with coronary, cerebrovascular, and peripheral arterial diseases, as well as deep veinous thrombosis in the general population [4, 5]. Three main pathophysiological changes intimately connected form the basis of HHcy-associated CVD [4, 29–33]: i) oxidative stress [4, 29–33], ii) rise in asymmetric dymethylarginine (ADMA) [31–33], iii) propensity for thrombosis [4, 7] (Fig. 2).

In situations of HHcy, homocysteine generates potent ROS free radicals through auto-oxidization of its highly active sulfhydryl group [29]. Continuous exposure of endothelial cells to higher homocysteine concentrations inhibits glutathione peroxidase, an enzyme that normally protects them against oxidative stress [4]. Together, these results indicate that HHcy induces vascular oxidative stress. Considering the physiological synthesis of nitric oxide (NO) by endothelial cells, vascular oxidative stress is responsible for reduced NO bioavailability [4, 29]. Endothelium-dependent NO levels may be further decreased by the reaction of NO with homocysteine at higher plasma homocysteine concentrations to form S-nitroso-homocysteine [4]. Of special relevance, reduced NO bioavailability subsequently induces endothelial dysfunction given the beneficial effects of the latter molecule (vascular tone regulation, inhibition of platelet activation, adhesion and aggregation, modulation of smooth cell proliferation and of endothelial-leukocyte interaction) [4, 29, 30]. In parallel, HHcy may dramatically increase the plasma concentration of ADMA-an endogenous nitric oxide synthase (NOS) inhibitor-by stimulation of its synthesis and inhibition of dimethylarginine dymethylaminohydrolase, the principal enzyme responsible for ADMA clearance [31]. ADMA is formed after proteolysis of proteins containing methylated arginine residues. Protein-arginine methylation is facilitated by protein methyltransferase enzymes which use S-adenosylmethionine (SAM) as the methyl donor group; SAM being released by adenosine triphosphate-activated L-methionine following homocysteine methylation [31, 32]. Through endothelial NOS inhibition, ADMA stimulates vascular oxidative stress, and consequently reduces NO bioavailability [4, 33]. Besides, ADMA depletes endothelial cells; thus worsening impairment of NO levels [33]. Taken together, HHcy induces endothelial dysfunction by reduction of NO levels through oxidative stress, formation of S-nitroso-homocysteine and raised ADMA production [4, 29–33].

Remarkably, atherogenesis is a complex process which likely starts by endothelial dysfunction [29–35], an“impaired endothelium-dependent blood-vessel dilation in response to a stimulus” [34]. Indeed, biological markers of endothelial dysfunction (Intracellular adhesion molecule-1, vascular cell adhesion molecule- 1, and P-selectin) are raised within the endothelium in the initial phase of atherosclerosis [34, 35]. Besides, these molecules may promote monocytes entry into the arterial wall where they become macrophages subsequently capturing oxidized low density lipoprotein (ox-LDL) molecules [34]. The resultant atherosclerotic plaque progression is under control of elevated cytokines released by inflammatory cells including ox-LDL fed macrophages termed “foam cells” [34]. Concurrent vascular smooth-muscle cell proliferation reinforces the plaque, and matrix metalloproteinases produced contributes to elastin and collagen breakdown within the arterial wall. Subsequent vessel fissuring is accompanied by entry of coagulation factors and platelet adherence that result in thrombosis, the late phase of atherosclerosis [35]. Noteworthy, HHcy stimulates endothelial dysfunction [4, 29–33], and lipid peroxidation [29–33] through oxidative stress and raised ADMA production. Furthermore, ADMA induces and amplifies macrophage transformation to foam cells within the arterial wall [4], and vascular smooth-muscle-cell proliferation [4]. Moreover, HHcy predisposes to thrombosis through platelet adhesion/activation and elevated production of clotting molecules (von Willebrand factor, factor V, protein C, tissue factor, lipoprotein (a) bounded to fibrin) [4] that can enter fissured arteries as well as impaired thrombolysis. Taken collectively, it is likely that HHcy directly and indirectly stimulates the atherosclerotic process throughout its development from the beginning with endothelial dysfunction to the end with thrombosis and resultant ischemia.

Beyond these major mechanisms, epigenetics, in particular DNA hypomethylation resulting from S-Adenosylhomocysteine accumulation over SAM may still facilitate atherogenesis by promotion of vascular smooth-muscle-cell proliferation through an oxidative stress dependent pathway. This can be seen in cases of mild to moderate HHcy [36]. Additional triggers of atherosclerosis in HHcy patients include traditional cardiovascular risk factors, especially arterial hypertension and a pro-atherogenic lipid profile with raised ox-LDL that leads to endothelial dysfunction and macrophage activation to foam cells within the arterial wall [7].

### In rheumatoid arthritis patients

RA is associated with a two-fold increased cardiovascular risk [1, 2] that persists after adjustment for traditional cardiovascular risk factors, suggesting the pivotal role of chronic inflammation [2]. More so, inflammatory biomarkers of cardiovascular risk less prevalent in the general population are more accurate in the course of RA [37, 38]. May it be the case for HHcy which is over two times more frequent in RA patients than in the general population (20–42 % versus 5–7 % for RA and general populations respectively) [29, 39]. However, only a few epidemiological studies have investigated the putative link between HHcy and CVD in RA populations (Table 1) [10, 21, 40–43]. In a prospective cohort including 235 RA patients prospectively followed up during a time frame of 6.5 year, fatal/non-fatal atherothrombotic events (myocardial infarction, ischemic heart disease, stroke, transient ischemic attack, deep vein thrombosis/pulmonary embolism) were all predicted by high homocysteine levels (OR = 1.96, 95 % CI 0.99–3.50, *p* = 0.05) after adjustment for age and sex [10]. In addition, high serum total homocysteine concentrations predicted cerebral white matter lesions-complications of cerebral microvascular disease- in Japanese RA women after adjustment for RA duration, serum triglycerides, serum high density lipoprotein cholesterol, and fasting plasma glucose (OR 1.35, 95 % CI: 1.12-1.63, *p* < 0.0001) [40].

**Table 1:** Studies investigating a link between hyperhomocysteinemia and cardiovascular diseases in rheumatoid arthritis patients

Author, year of publication, location	Population	Study design	Cardiovascular outcome	Key findings	Comments
Berglund et al., 2009, Sweden [10]	235 RA patients; 68 males (52 ± 16 years)^a^ and 166 females (46 ± 16 years)^a^	Prospective cohort study	Atherothrombotic events	HHcy levels adjusted for age and sex were a significant predictor of atherothrombotic events (OR = 1.96, 95 % CI 0.99–3.50, *p* = 0.05).	When adjusted for hypertension alongside age and sex, The predictive value of HHcy was reduced (OR = 1.84, 95 % CI 0.92–3.69, *p* = 0.084] for hypertension and OR = 1.80, 95 % CI 0.90–3.59, *p* = 0.096 for diabetes mellitus).
Anan et al., 2009, Japan [40]	25 RA women with WML (61 ± 6 years)^a^ and and 40 RA women without WML (60 ± 7 years)^a^	Case–control study	WML	HHcy independently predicted WML (OR 1.35, 95 % CI: 1.12-1.63, *p* < 0.0001).	Adjusted for the duration of RA, triglyceride, HDL, FPG
Dala et al., 2012, Egypt [41]	180 RA patients with no history of IHD	Cross-sectional study	SIHD	Serum Hcy was significantly higher in patients with SIHD as compared to those without (*p* < 0.001)	
Chung et al., 2005, USA [42]	141 patients with RA (median age 54, IQR 46–64), 68 % of females; and 86 controls without RA (median age 52, IQR 44–59), 65.1 % of females	Case–control study	Coronary-artery atherosclerosis (calcification)	In unadjusted comparisons, HHcy was more common in RA patients with coronary-artery calcification than in those without.	After adjusting for age and sex, the association was no more significant
Cisternas et al., 2002, Chili [43]	54 RA patients (51 ± 13 years)^a^; and 32 age and sex matched healthy controls	Case–control study	History of CVD	There were higher Hcy plasma levels in RA patients with a history of CVD than in those without.	
Seriolo et al., 2001, Italy [21]	168 female RA women with WML (cases), 50 ± 10 years^a^; and 72 age and sex matched healthy controls (52 ± 9 years)^a^.	Case–control study.	History of thrombotic events	Plasma levels of hcy in aPL antibody-positive patients with thrombosis were found to be significantly higher than in aPL antibody-negative RA patients without thrombosis (*p* < 0.001)	Adjusted for the duration of RA, triglyceride, HDL-Cholesterol, fasting plasma glucose

*RA* rheumatoid arthritis, *SD* standard deviation, *OR* odds ratio, *95* % *CI* 95 % confidence interval, *FPG* fasting plasma glucose, *HHcy* hyperhomocysteinemia, *White matter lesions* (*WML*) are considered as ischemic complications of cerebral microvascular disease, and important prognostic factor for the development of stroke, *HDL* high density lipoprotein cholesterol, *ECG* electrocardiogram, *Hcy* homocysteine, *CVD* cardiovascular disease, *aPL-positive* antiphospholipid antibody positive, *aPL-negative* antiphospholipid antibody negative, *silent ischemic heart disease (SIHD)* was diagnosed as ischemia on stress test in the absence of angina and/or ECG changes of either a bundle branch block or ST segment abnormality consistent with, *IHD* ischaemic heart disease, *IQR* interquartile range

^a^Age is expressed as mean ± standard deviation

The pathophysiology of HHcy-derived CVD in RA patients is largely uncharacterized. Nevertheless, indirect evidence from relevant observations in the general population together with the crucial role of inflammation push forward our understanding of the several derangements that may interact to cause or exacerbate it [6, 11, 12, 14, 21, 22, 36–38, 44–51]. They include: i) oxidative stress [12, 44] ii) chronic inflammation and immune activation [11, 14, 37, 38, 45] iii) propensity for a pro-atherogenic lipid profile [44–47] iv) poor disease status and severe radiological damage [6, 12, 38, 44] v) thrombophilia [21, 22, 38] vi) increase of plasma ADMA levels [14, 48] vii) osteoprotegerin [49] viii) genetic and epigenetic factors [36, 50, 51]. For details, see Table 2.

**Table 2:** Putative mechanisms and factors involved in HHcy-related CVD in RA

Mechanism/factor contributing to HHcy-dependent CVD	Comment
Oxidative stress [12, 44]	Induction of endothelial dysfunction and atherosclerosis throughout impaired NO availability, increased lipid peroxidation and activation of NF-ƙB by Hcy-derived ROS, thus of inflammatory cascade
Chronic inflammation and immune activation [12, 14, 37, 38, 45]	Inflammatory biomarkers (IL-1, IL-6, TNF-α, CRP) are related with impaired NO availability, endothelial dysfunction, arterial stiffness, and a prothrombotic status
	Autoantibodies against peptides modified by homocysteine-thiolactone can worse inflammation and hence maintain an increased cardiovascular risk [14].
Pro-atherogenic lipid profile [44–47]	Healthy HDL molecules are tighly linked to PON1, an antioxidant enzyme which is diminished in RA
	The remaining low HDL is pro-inflammatory and can no longer counteract LDL oxidation
	Ox-LDL activates endothelium and favors atherosclerosis
High disease activity and severe radiological damage [6, 12, 14, 38, 44]	They have been associated with HHcy and both reflect chronic inflammation.
	The proatherogenic profile in RA highly depends on disease activity
Antiphospholipid autoantibodies and other thrombogenic molecules [21, 22, 38]	The prevalence of aPL is high (28 %) in RA
	In aPL-positive RA patients, aPL may interact with Hcy to increase the risk of thrombosis
	Several procoagulant molecules are correlated with endothelial dysfunction and thrombosis
ADMA [14, 48]	Increased plasma ADMA is independently associated with carotid atherosclerosis in RA
	ADMA may cause endothelial dysfunction since higher serum levels have been associated with a decreased CFR in RA
Osteoprotegerin^a^ [49]	OPG is increased in RA and is independently associated with carotid artery calcification in RA, probably to counteract increased RANKL production
	Considering that HHcy has been associated with OPG in RA, and with respect to Hcy’s ability to stimulate RANKL, it is possible that OPG is a marker of Hcy-mediated CVD in RA.
Epigenetic and genetic factors [36, 50, 51]	Interaction of HHcy with NFKB1-94ATTG ins/del polymorphism constitutively activated in RA patients to accentuate immune responses and that predispose RA patients to subclinical and accelerated atherosclerosis
	RA is characterized by DNA hypomethylation which is implicated in atherosclerosis in the general population. It can be hypothesized that it may partly explain CVD in relation to HHcy in RA patients, but this is yet to be ascertained

*CVD* cardiovascular diseases, *NO* nitric oxide, *NF- ƙB* Nuclear Factor ƙappa B, *ROS* reactive owxygen species, *IL-1* interleukin 1, *IL-6* interleukin 6, *TNF-α* tumor necrosis factor alpha, *CRP C* reactive protein, *IFN-Ɣ,* interferon gamma, HDL high density lipoprotein cholesterol, *LDL* low density lipoprotein cholesterol, *PON1* paroxonase type 1, *RA* rheumatoid arthritis, *ox-LDL* oxidized low density lipoprotein cholesterol, *IMT* increased media-thickness, *HHcy* hyperhomocysteinemia, *aPL* antiphospholipid, *ADMA* asymmetric dymethylarginine, *Hcy* homocysteine, *CFR* coronary flow reserve, *Osteoprotegerin* ^a^ soluble glycoprotein which is an inibitor of the receptor activator of nuclear factor-ƙB (RANKL), *OPG* osteoprotegerin, *CVD* cardiovascularv diseases, *DNA* deoxyribonucleic acid

Unlike in the general population where oxidative stress, ADMA and a prothrombotic status are the main determinants of HHcy-dependent CVD, oxidative stress and long-term shift in immuno-inflammatory activation are central for the occurrence and worsening of HHcy-mediated CVD in RA [8]. In fact, excess ROS free radicals released by homocysteine oxidation can enhance the Nuclear Factor ƙappa B (NF-ƙB) activity already upregulated in RA patients considering NF-ƙB as the master regulator of expression of inflammation genes [11, 12, 50]. Resultantly, pro-inflammatory biomarkers are excessively released into the circulation, perpetuating immuno-inflammatory activation [11]. The high inflammatory burden collectively with oxidative stress promote excess lipid peroxidation to form ox-LDL [38, 50], markers of subclinical atherosclerosis in RA [47]. Lipid peroxidation is sustained and aggravated by a high disease activity, and activates the endothelium. Besides, ox-LDL molecules are captured by macrophages, becoming foam cells that maintain the atherosclerotic plaque progression through persistent cytokine production [6, 38]. Furthermore, TNF-α promotes vascular smooth-muscle-cell proliferation. Moreover, IL-1, IL-6 and TNF-α are procoagulant since they upregulate tissue factor (that can enter into the injured arterial wall) and trigger endothelial cells to become prothrombotic (alongside oxidative stress) [37, 38]. Additional prothrombotic markers in the RA population such as aPL could interact with homocysteine to cause thrombotic events [21, 22]. Hence, thrombosis-the late phase of atherosclerosis- might occur as a result of oxidative stress together with pro-inflammatory biomarkers’ activity and aPL. In brief, the bi-directional link between homocysteine and immuno-inflammatory activation [8, 11] well illustrates how HHcy could contribute to a persistent worsened high risk for CVD in RA patients.

## Folate supplementation, “a therapeutic potential preventive strategy for cardiovascular outcomes through homocysteine lowering and blockade”

### In the general population

Dozens of studies assessing the benefits of folic acid supplementation on vascular disease have been conducted to date, initially on animal models. El-Swefi and collaborators observed a significant increase of plasma NO concentration in ovariectomized rats co-treated with folic acid and estradiol compared with ovariectomized and estradiol-treated groups (15.5 ± 2.8 μmol/l vs 9.4 ± 2.5 μmol/l and 15.5 ± 2.8 μmol/l vs 12.2 ± 3 μmol/l respectively; *p* < 0.05 for all comparisons). Additionally, folic acid co-treatment with estradiol resulted in 23 % inhibition of copper-induced lipoprotein oxidation in comparison with the ovariectomized non-treated group [52]. Likewise, addition of folic acid to a homocystine-rich diet concomitantly reduced the occurrence of damaged cerebral vessels and raised glucose transporter protein-1 (a marker of cerebral endothelial dysfunction since it facilitates glucose transport across blood brain barrier endothelium) plasma concentration in male Sprague–Dawley rats after eight weeks [53]. Moreover, vitamin therapy (B2 or B6 plus B9) intriguingly inhibited neurologic signs of ischemic cerebral attack (unbalance, ataxia, and convulsions) in spontaneously hypertensive stroke-prone rats [54]. Note that all those changes were paralleled by homocysteine lowering [52–54]. Taken together, folic acid supplementation and subsequent homocysteine lowering might inhibit oxidative stress (thus reducing lipid peroxidation) and reverse endothelial dysfunction, with a net benefit on cerebral vessels.

Along these lines, there is compelling evidence from general population studies that folate supplementation reduces the risk of incident cerebrovascular events (ischemic/hemorrhagic) [55, 56]. Of particular relevance, a recent meta-analysis of 26 randomized double-blind placebo-controlled studies including 58,804 subjects reported a strong trend in the reduction of future stroke risk of 7 % (RR 0.93, 0.86 to 1.00; *p* = 0.05) with folic acid supplementation [55]. Consistent with this finding, a large double blinded clinical trial published this year observed a reduced risk of first stroke attributed to the folic acid-enalapril combination therapy as compared to enalapril administered alone (2.7 % of participants in the enalapril–folic acid group vs 3.4 % in the enalapril alone group; hazard ratio 0.79; 95 % CI, 0.68-0.93) [57]. Low baseline folate levels and high baseline plasma homocysteine were the main tenants of this benefit [55, 56]. Contrariwise, folic acid supplements do not prevent incident coronary heart disease (CHD). This has been suggested by a meta-analysis of large-scale randomized placebo-controlled clinical trials held over a five-year period, and involving 35,603 participants with no heterogeneity between trials [58].

Furthermore, folic acid supplementation does not appear beneficial for secondary prevention of CVD [59–61]. In particular, recurrent stroke cannot be prevented with folate therapy [59]. Additionally, the Vitamins and Thrombosis (VITRO) randomized placebo-controlled double blind trial failed to demonstrate a reduced risk of secondary deep veinous thrombosis and pulmonary embolism after B vitamin therapy [60]. However, it is still inconclusive whether or not folic acid is effective for the prevention of CHD in persons with previous CVD [59, 61]. The meta-analysis by Mei and collaborators reported a lack of efficacy of folic acid supplementation in the prevention of recurrent CHD (0.94 [0.85–1.04]) [59], same as Qin and collaborators [61]. Nevertheless, these last authors whose meta-analysis detected a substantial heterogeneity among studies observed a trend towards reduced coronary revascularization risk when folic acid and moderate B_6_ were co-supplemented. Therefore, the effect of folic acid supplementation on CHD is worthy of definite clarifications.

In total, with the exception of primary prevention of cerebrovascular events [52–57], current knowledge does not support the systematic supplementation of folic acid for CVD primary or secondary prevention in the general population [58–61]. It has been speculated that HHcy preferentially targets cerebral microvasculature [55]. This may explain why folic acid therapy works only on primary prevention of stroke. In addition, it is possible that cardiovascular changes progress independently of homocysteine level once established [62]. This observation can help understand why folic acid supplementation does not effect on the prevention of recurrent CVD. But why is there a disappointment with vitamin therapy between primary and secondary preventions of stroke? Folic acid effectiveness might be increased in the earliest phase of cerebrovascular disease rather than in the late phase [61]. Following this observation, a meta-analysis of 14 randomized double-blind placebo-controlled trials including 732 people reported an improvement of endothelial dysfunction as measured by vessel flow mediated dilation after four weeks of folic acid supplementation, and this effect seemed independent of serum homocysteine reduction [30]. These changes were paralleled by a sharp reduction of total homocysteine plasma concentration. The findings of this meta-analysis further support reparation of damaged endothelium by folic acid treatment independently of plasma homocysteine reduction.

### In rheumatoid arthritis

In spite of the acknowledged cardiovascular derangements of HHcy in RA patients [10, 21, 40–43], we still lack data regarding the effectiveness of homocysteine-lowering strategies for CVD prevention in this population. Yet, almost all prospective studies have clearly demonstrated a reduction of all-cause CVD morbidity and mortality associated with methotrexate treatment [63–65], and this benefit is thought to be partly confounded by folic acid supplementation which lowers methotrexate-induced HHcy [23, 25–28]. Still, the methotrexate-folic acid combination resulted in a reduced mortality hazard ratio of 0.2, compared with 0.5 for methotrexate alone in a RA cohort probably because of homocysteine lowering via folic acid supplements [66]. Moreover, a follow-up study of RA patients showed a rise in plasma homocysteine after a low-dose methotrexate of 7.5-10 mg/week. Most importantly, homocysteine levels were negatively correlated with folic acid co-treatment [67]. Contrasting with sparse homocysteine-lowering published studies in RA patients is their high cardiovascular risk.

It is remarkable that RA patients have a 60 % increased risk of death from myocardial infarction and stroke compared with the general population [3], and a 48 % increased risk of incident myocardial infarction [68]. Folic acid depletion and subsequent HHcy-common in RA patients- partly explain this trend via excess immuno-inflammatory activation [7, 11, 12, 38, 45]. Furthermore, variations of plasma homocysteine may significantly predict atherosclerosis progression in RA patients [69]. Therefore, homocysteine-lowering strategies might be suitable to curtail the CVD ‘epidemic’ in RA populations.

Learning from general population and animal studies [55–57] and more than in the general population, folic acid supplements could reduce incident cerebrovascular events in the RA population as well if we consider their high propensity for HHcy and folate deficiency [55–57]. This hypothesis can be further supported by the central role of oxidative stress in HHcy-mediated cerebrovascular disease regardless of populations and species [4, 7, 52–54] together with the likelihood of folic acid to reverse oxidative stress both independently of and via homocysteine lowering [56, 69]. However, it is questionable whether or not the likely impact of folate therapy on the cerebral microvasculature of RA patients extends to coronary and other peripheral vessels. Extrapolating from general population meta-analysis addressing the benefits of vitamin therapy on extracerebral vessels [59, 61], the answer is a priori negative. Nonetheless, the positive response cannot be completely ruled out. Indeed, a meta-analysis of 10 randomized double-blind placebo-controlled clinical trials totalizing 2052 subjects reported a significant decrement of carotid intima-media thickness (CIMT) in relation with folic acid supplementation, and this benefit was greater among high CVD risk individuals, with larger homocysteine reduction and higher baseline CIMT values being strongest predictors [70]. Notably, CIMT is a reliable marker of atherosclerosis progression and a strong predictor of future cardiovascular events including CHD in both general and RA populations [71, 72]. Besides, RA patients are high CVD risk subjects [1, 3, 6, 68] with higher baseline CIMT values [72] that steeply rise partly because of HHcy [69]. Thus, it can be speculated that folic acid supplementation may also significantly reduce or delay atherosclerosis progression, consequently preventing all-cause CVD in RA patients.

Overall, folic acid supplementation is an effective anti-oxidant therapy which could directly and indirectly limit immuno-inflammatory activation and lipid peroxidation as well as repair endothelial damage through oxidative stress antagonism in RA patients. Furthermore, it may positively impact on atherosclerosis progression via plasma homocysteine decrement. In light of these observations, we put forward the hypothesis that it may offer therapeutic potentials for CVD prevention in RA patients in the near future. Large-scale and long-term clinical trials examining the impact of folic acid supplementation on CVD in RA populations will be very informative in this context. In particular, numerous questions should be clarified by the several randomized controlled trials underway [73]. Is folate supplementation really associated with CVD risk reduction in RA patients with or without CVD? To which extent can folate supplementation be beneficial for CVD prevention in RA patients? What is the precise mechanism underlying HHcy-related CVD in RA patients? What are optimal dosage and frequency for folate administration? If correct, our hypothesis could have sizeable public health implications in the issue of CVD in RA patients. While awaiting, CVD prevention through systematic supplementation with folic acid is desirable in both RA patients at high risk for HHcy/folic acid deficiency (e.g., those taking antifolate agents such as methotrexate and sulfasalazine, those who have a folate-deficient diet) and those with ascertained HHcy. A weekly folic acid dose of five milligrams has been proposed for those taking methotrexate [10], while other subjects might be given the current recommended nutrient intake for folates (1.4 mg week^−1^) [9] in absence of a specific evidence-based recommended dose.

## Conclusion

CVD may be prevented in RA patients via folic acid supplementation that potentially lowers plasma homocysteine levels and inhibits/repairs its cardiovascular health hazards. However, large-scale and long-term homocysteine-lowering clinical trials with folate are warranted to conclude on causality of the association between HHcy and CVD in RA and to definitely clarify conditions of folate supplementation for a cardiovascular issue. Anyway, systematic supplement with folic acid of all RA patients at high risk for folic acid deficiency/ HHcy or those with confirmed HHcy is desirable in order to prevent CVD.

## Abbreviations

ADMA: Asymmetric dymethylarginine
aPL: Antiphospholipid
CHD: Coronary heart disease
CIMT: Carotid intima-media thickness
CVD: Cardiovascular diseases
DMARDs: Disease-modifying antirheumatic drugs
DNA: Deoxyribonucleic acid
HHcy: Hyperhomocysteinemia
IFN-γ: Interferon gamma
IL-1: Interleukin 1
IL-6: Interleukin 6
MTHFR: Methylenetetrahydrofolate reductase
NF-ƙB: Nuclear factor kappa B
NO: Nitric oxide
NOS: Nitric oxide synthase
OR: Odds ratio
ox-LDL: Oxidized low density lipoprotein
ROS: Reactive oxygen species
SAM: S-adenosylmethionine
TNF-α: Tumor necrosis factor alpha
